# A Minimal Subset of Seven Genes Associated with Tumor Hepatocyte Differentiation Predicts a Poor Prognosis in Human Hepatocellular Carcinoma

**DOI:** 10.3390/cancers13225624

**Published:** 2021-11-10

**Authors:** Matthis Desoteux, Corentin Louis, Kevin Bévant, Denise Glaise, Cédric Coulouarn

**Affiliations:** 1 Inserm, Univ. Rennes, UMR1242, Chemistry Oncogenesis Stress Signaling (COSS), 35042 Rennes, France; matthis.desoteux@univ-rennes1.fr (M.D.); corentin.louis@univ-rennes1.fr (C.L.); kevin.bevant@univ-rennes1.fr (K.B.); 2 Inserm, Univ. Rennes, UMR991, Liver Metabolisms and Cancer, 35043 Rennes, France; denise.glaise@inserm.fr

**Keywords:** hepatocellular carcinoma, prognosis, signature, differentiation, overall survival, integrative transcriptomics

## Abstract

**Simple Summary:**

Liver cancer is one of the most commonly diagnosed cancers worldwide and the fourth leading cause of cancer-related deaths. Hepatocellular carcinoma (HCC) accounts for at least 80% of all malignant liver primary tumors. A better characterization of molecular mechanisms underlying HCC onset and progression may lead to discover new therapeutic targets and biomarkers. In this study, we performed an integrative transcriptomics analysis to evaluate the clinical relevance of genes associated with hepatocyte differentiation in human HCC. The HepaRG cell line model was used to define a gene expression signature reflecting the status of tumor hepatocyte differentiation. This signature was able to stratify HCC patients into clinically relevant molecular subtypes. Then, a minimal subset of seven differentiation-associated genes was identified to predict a poor prognosis in several cancer datasets.

**Abstract:**

Hepatocellular carcinoma (HCC) is a deadly cancer worldwide as a result of a frequent late diagnosis which limits the therapeutic options. Tumor progression in HCC is closely correlated with the dedifferentiation of hepatocytes, the main parenchymal cells in the liver. Here, we hypothesized that the expression level of genes reflecting the differentiation status of tumor hepatocytes could be clinically relevant in defining subsets of patients with different clinical outcomes. To test this hypothesis, an integrative transcriptomics approach was used to stratify a cohort of 139 HCC patients based on a gene expression signature established in vitro in the HepaRG cell line using well-controlled culture conditions recapitulating tumor hepatocyte differentiation. The HepaRG model was first validated by identifying a robust gene expression signature associated with hepatocyte differentiation and liver metabolism. In addition, the signature was able to distinguish specific developmental stages in mice. More importantly, the signature identified a subset of human HCC associated with a poor prognosis and cancer stem cell features. By using an independent HCC dataset (TCGA consortium), a minimal subset of seven differentiation-related genes was shown to predict a reduced overall survival, not only in patients with HCC but also in other types of cancers (e.g., kidney, pancreas, skin). In conclusion, the study identified a minimal subset of seven genes reflecting the differentiation status of tumor hepatocytes and clinically relevant for predicting the prognosis of HCC patients.

## 1. Introduction

Liver cancer is one of the most commonly diagnosed cancers worldwide (841,000 cases) and the fourth leading cause of cancer-related deaths (782,000 cases) [1]. Both the incidence and the mortality of liver cancer have increased over the last two decades. Hepatocellular carcinoma (HCC) accounts for at least 80% of all malignant liver primary tumors. HCC frequently occurs in a background of fibrotic and/or cirrhotic liver and is usually asymptomatic during the early phases of carcinogenesis. Thus, most of the patients are diagnosed with advanced stage HCC, which limits the therapeutic options and their efficacy [2]. Tumor heterogeneity in advanced HCC also impedes the development of effective treatments. Genome-scale studies at the DNA level (e.g., mutation analysis, epigenetic profiling) established the landscape of molecular alterations occurring in HCC. Mutations in the *TERT* promoter and in *TP53*, and *CTNNB1* genes were identified as the most frequent driver mutations in HCC [3].

Gene expression profiling studies at the RNA level defined key genes and pathways commonly altered in HCC [4] and identified clinically relevant tumor subtypes [5,6,7]. By integrating these studies, a general consensus on the molecular classification of human HCC has been achieved [5]. Thus, HCC are classified into two main molecular classes: a proliferation class and a non-proliferation class. The proliferation class includes aggressive and poorly differentiated HCC associated with frequent signs of vascular invasion. These HCC are more frequently mutated for *TP53* [8]. This proliferation class is further subdivided into a “WNT-TGFβ” subclass defined by the activation of WNT and TGFβ pathways and an exhausted immune response, and a “progenitor” subclass defined by the expression of progenitor markers [6,9,10]. In contrast, the non-proliferation class includes more differentiated HCC with hepatocyte-like features, the up-regulation of liver-specific genes, and a better prognosis [5,8]. A gene regulatory network linked with β-catenin dysfunction and dedifferentiation was found to be negatively correlated with HCC prognosis [11]. It was also reported that the overexpression of hepatocyte nuclear factor-1 alpha (HNF1A), a key transcription factor driving hepatocyte differentiation, inhibited HCC growth in mice [12].

Altogether, unsupervised gene expression profiling and supervised functional studies established a functional link between hepatocyte dedifferentiation, HCC progression and a poor clinical outcome, suggesting that dedifferentiation markers could be clinically relevant to predict HCC prognosis [6,7,8,13]. However, no experimentally validated signature including an exhaustive list of genes linked with differentiation has been reported so far. Most of the prognostic signatures derived from transcriptomics studies consist of hundreds of genes, some of them related to differentiation, rendering a routine clinical implementation challenging [14]. Here, we used the HepaRG cell line model cultured under well-controlled conditions recapitulating tumor hepatocyte differentiation [15,16,17] combined with an integrative transcriptomics approach [18,19] to identify a minimal subset of seven genes reflecting the differentiation status of tumor hepatocytes and clinically relevant for predicting overall survival in independent cohorts of patients with HCC.

## 2. Materials and Methods

### 2.1. Culture of HepaRG Cells

HepaRG cells were cultured as previously described [18]. Briefly, cells were grown in William’s E medium supplemented with 10% FBS, 100 U/mL penicillin, 100 mg/mL streptomycin, 5 mg/mL insulin, and 50 mmol/L hydrocortisone hemisuccinate. Differentiation of HepaRG from progenitors to mature well-differentiated hepatocytes was achieved within 4 weeks by culturing the cells in the supplemented medium in presence of 2% dimethyl sulfoxide (DMSO) for the last 2 weeks. All experiments, hereinafter referred to as progenitor HepaRG, were carried out using cells isolated 3 days after plating, while experiments referred to as differentiated HepaRG were carried out using hepatocytes selectively isolated by mild trypsinization from DMSO-treated cultures 4 weeks after the initial plating. All cell cultures were conducted at 37 °C in a 5% CO_2_ atmosphere. Independent culture experiments were carried out in triplicate.

### 2.2. Gene Expression Profiling

Total RNA was purified from cells at 80% confluence with a RNeasy kit (Qiagen). Genome-wide expression profiling was conducted using the low-input Quick Amp Labeling Kit and human SurePrint G3 8 60 K pangenomic microarrays (Agilent Technologies) as previously described [4]. Starting from 150 ng total RNA, amplification yield was 9.7 ± 0.6 µg cRNA and specific activity was 20.3 ± 1.3 pmol Cy3 per µg cRNA. Gene expression data were processed using the Feature Extraction and GeneSpring software (Agilent Technologies) and further analyzed using R-based ArrayTools. Clustering analysis was carried out using Cluster 3.0 and TreeView 1.6 using uncentered correlation and average linkage options. MIAME compliant microarray data have been deposited into the Gene Expression Omnibus (GEO) database (GSE181963).

### 2.3. Data Mining and Integrative Transcriptomics

Gene annotation was based on Gene Ontology terms and enrichment for specific biologic functions or canonical pathways was evaluated using FuncAssociate 2.0 [20]. Gene set enrichment analysis (GSEA) was conducted by using the Java tool developed at the Broad Institute (Cambridge, MA, USA) as previously described [21]. Unsupervised GSEA was carried out with the whole C2 collection of curated gene sets from the molecular signatures database (MSigDB). Enrichment score was determined after 1000 permutations. Integration of genomic data was conducted as previously described [19] using publicly available gene expression data sets downloaded from GEO. Survival analysis was conducted using the TCGA datasets (https://portal.gdc.cancer.gov, accessed on July 2021).

### 2.4. Real-Time Reverse Transcriptase PCR

Expressions of relevant genes were measured by quantitative real-time reverse transcriptase PCR (qRT-PCR), as previously described [22]. Quantitative analysis of PCR data was conducted with the 2ΔΔCt method using glyceraldehyde-3-phosphate dehydrogenase (GAPDH) Ct values for normalization. Melting analysis was conducted to validate the specificity of PCR products. PCR and gene expression profiling experiments were conducted using RNA extracted from independent cultures (*n* = 3).

### 2.5. Statistical Analysis

Data are presented as the mean ± standard deviation (SD). Statistical analyses were performed using R-3.5.1 and GraphPad Prism 7.0. For gene expression profiling, differentially expressed genes were identified by a 2-sample univariate t test and a random variance model (*p* < 0.01; false discovery rate < 1%) as described [23]. Permutation *p* values for significant genes were computed on the basis of 10,000 random permutations. For group comparison of quantitative variables, a Mann–Whitney test was applied. Categorical data were analyzed by chi-squared testing. The cumulative survival rate was estimated by the Kaplan–Meier method and the survival curves were compared with the log-rank test. *p* values < 0.05 were considered as significant statistical differences.

## 3. Results

### 3.1. Flowchart of the Study Design

**Figure 1:**
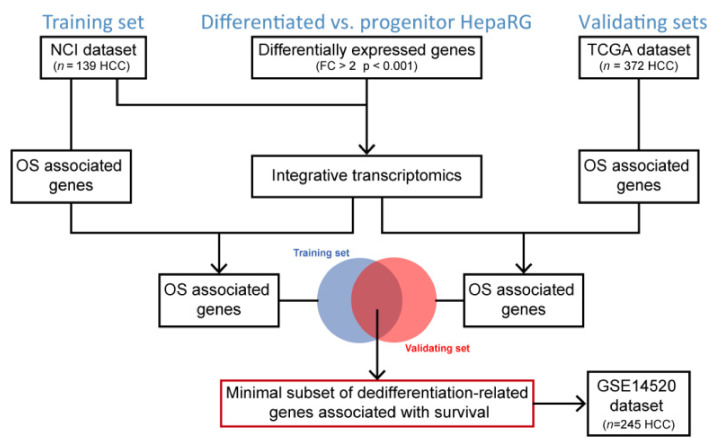
Flowchart of the study design. OS, overall survival.

Gene expression profiling was performed to identify genes significantly differentially expressed between differentiated and progenitor HepaRG cells (Figure 1). Then, an integrative transcriptomics analysis was conducted using publicly available human HCC gene expression datasets downloaded from GEO to evaluate the clinical relevance of the hepatocyte differentiation signature derived from HepaRG, as previously described [18]. The so-called NCI dataset (GSE1898 and GSE4024) was used as a training set (*n* = 139 HCC) and the TCGA RNA-seq dataset (https://portal.gdc.cancer.gov, accessed on July 2021) was used as a validating dataset (*n* = 372 HCC), as well as the GSE14520 dataset (*n* = 245 HCC). Kaplan–Meier plots and log-rank statistics were used to identify genes associated with overall survival (OS). Merging the OS-associated genes from the training and validating datasets was performed to identify a minimal subset of differentiation-related genes predicting OS in patients with HCC (Figure 1).

### 3.2. Differentiation-Associated Genes in HepaRG Cells

**Figure 2:**
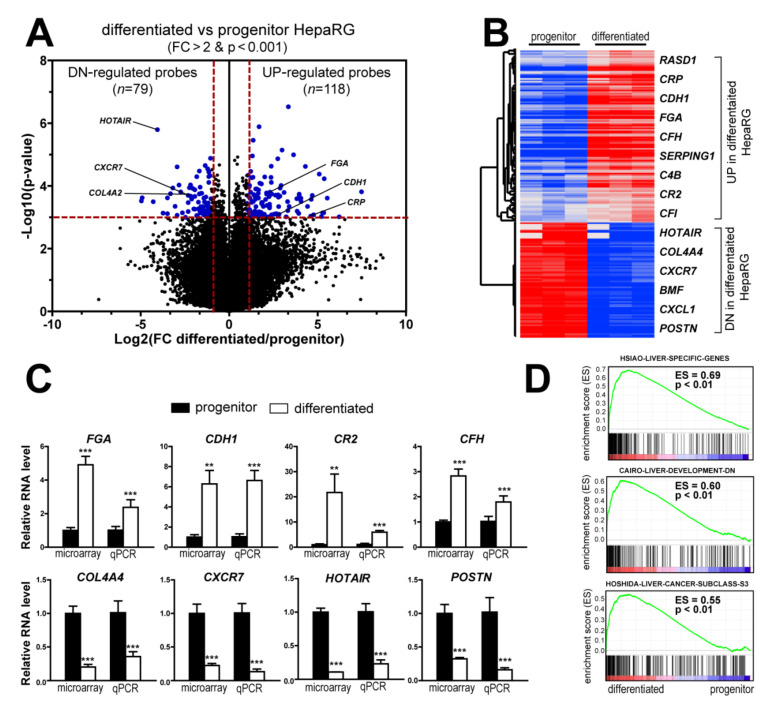
Differentiated and progenitor HepaRG cells exhibit distinct gene expression profiles related to hepatocyte differentiation. (**A**) Volcano plot of genes differentially expressed between differentiated and progenitor HepaRG cells. Differentiated HepaRG cells display a specific signature of 118 probes (105 genes) up-regulated and 79 probes (69 genes) down-regulated. Horizontal dashed red line: *p* < 0.001. Vertical dashed red lines: FC > 2. (**B**) Hierarchical clustering analysis and examples of genes up- and down-regulated in differentiated and progenitor HepaRG cells. The complete list of probes and genes is provided in the Supplementary Table S1. (**C**) Gene expression analysis of representative genes differentially expressed in differentiated and progenitor HepaRG cells, as determined by microarray and qRT-PCR. Statistical analysis was performed by a Mann–Whitney test (** *p* < 0.01, *** *p* < 0.001, *n* ≥ 3). (**D**) GSEA analysis using the gene expression profiles of differentiated (left side) and progenitor (right side) HepaRG cells. GSEA highlights a significant enrichment (*p* < 0.01) of several liver-related signatures, including the so-called HSIAO_LIVER_SPECIFIC_GENES [26], CAIRO_LIVER_DEVELOPMENT_DN [27] and HOSHIDA_LIVER_CANCER_SUBCLASS_S3 [6] signatures.

By using stringent statistical criteria (*p* < 0.001 and fold change FC > 2), the volcano plot analysis identified 197 probes, corresponding to 174 non-redundant and well-annotated genes differentially expressed between differentiated and progenitor HepaRG cells. This signature included 105 up- and 69 down-regulated genes in differentiated HepaRG cells as compared to HepaRG progenitors (Figure 2A). The clustering analysis highlighted genes related to liver-specific metabolisms (Figure 2B and Table S1). Thus, genes associated with the complement and coagulation pathways (e.g., *C2*, *C4B*, *CFH*, *CR2*, *PROC*) or encoding secreted proteins involved in the response of the liver to acute systemic inflammation (e.g., *A2M*, *CRP*, *FGA*, *ORM1*, *SAA2*, *SERPINA1*) [24,25] were significantly induced in differentiated HepaRG cells. Similarly, genes encoding members of the cytochrome P450 superfamily (e.g., *CYP1A1*, *CYP8B1*) involved in drug metabolism and lipid synthesis were more expressed in differentiated HepaRG, as well as genes encoding alcohol-metabolizing enzymes (e.g., *ADH1A*, *ALDH2*) and nuclear receptors regulating xenobiotic metabolism (e.g., *NR1I3*). Other genes more expressed in differentiated HepaRG cells were involved in glutathione (e.g., *GPX3*) and amino acid (e.g., *PAH*, *PIPOX*) metabolisms, ketogenesis (e.g., *HMGCS2*) or encoding cell junctions (e.g., *CDH1*, *TJP3*), aquaporins (e.g., *AQP9*, *AQP11*) and transmembrane proteins (e.g., *SLC17A1*, *SLC22A23*), all of them reflecting well-differentiated functional hepatocytes. Conversely, the genes more expressed in HepaRG progenitors were associated with cell cycle, proliferation and apoptosis (e.g., *CDK20*, *NUAK1*, *BMF*, *CASP7*), hypoxia (e.g., *CITED2*), chemoattraction (e.g., *CXCL1*, *CXCR7*) and angiogenesis (e.g., *EDIL3*, *NRP1*). Interestingly, several genes were known to be regulated by the TGFβ pathway (e.g., *BMF*, *COL4A4*) and/or expressed in several cancers (e.g., *HOTAIR*, *MRC2*, *POSTN*, *SPARC*). Next, the expression of eight representative genes were evaluated by qRT-PCR (Figure 2C), including four genes more expressed in differentiated HepaRG cells (*FGA*, *CDH1*, *CR2* and *CFH*) and four genes more expressed in HepaRG progenitors (*COL4A4*, *CXCR7*, *HOTAIR* and *POSTN*). The expression of all genes was validated (Figure 2C). Further supporting the gene selection, GSEA demonstrated that well-curated liver-related gene signatures were significantly enriched in the gene expression profiles of differentiated HepaRG cells (Figure 2D), including liver-specific genes [26] as well as gene sets associated with liver development [27] and well-differentiated HCC tumors (e.g., the so-called HCC molecular subclass S3 defined by Hoshida and colleagues [6]). Altogether, these data validate the gene expression signature established in HepaRG cells and suggest that it could be relevant in vivo, both in mouse liver development and in human liver carcinogenesis.

### 3.3. HepaRG-Derived Signature Recapitulates Relevant Stages of Mouse Liver Development

**Figure 3:**
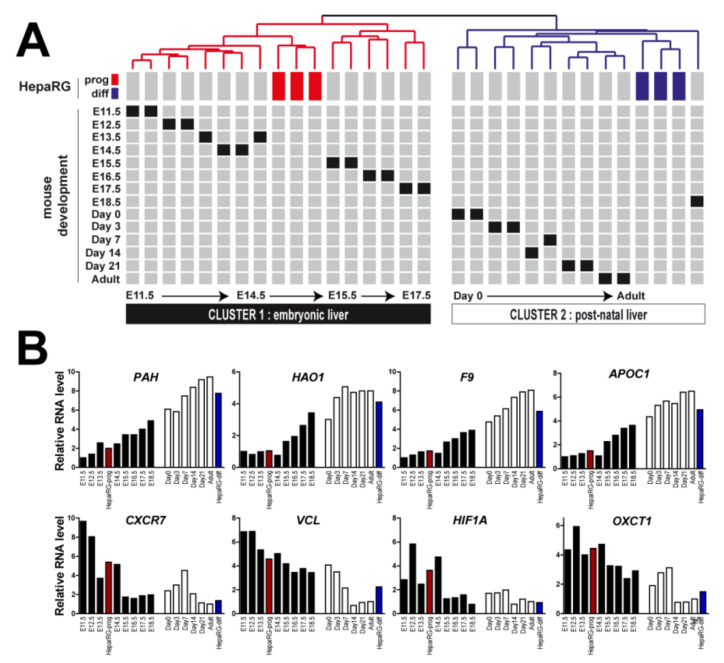
Differentiated and progenitor HepaRG gene profiles recapitulate relevant stages of mouse liver development. (**A**) Clustering of mouse and human samples based on the expression of orthologous genes identified from the signature of genes differentially expressed between differentiated and progenitor HepaRG cells. Data are presented in a matrix format in which rows and columns represent the position of each sample within the dendrogram. Mouse gene expression profiling was performed between E11.5 to adult stage. Clustering analysis identified two major clusters: cluster 1, which includes progenitor HepaRG cells (red boxes), is associated with embryonic mouse liver samples and cluster 2, which includes differentiated HepaRG cells (blues boxes), is associated with post-natal mouse liver samples. (**B**) Examples of the expression of developmentally regulated genes. Red bars: progenitor HepaRG cells; blue bars: differentiated HepaRG cells; black bars: embryonic stages in mice (from E11.5 to E18.5, left to right); white bars: post-natal stages in mice (from Day0 to adult stage, left to right).

To determine the in vivo relevance of the gene expression profiles derived from HepaRG cells (differentiated versus progenitor), we integrated them with the multi-stage gene expression profiles established in the course of mouse liver development [28]. The GSE13149 dataset reports transcriptomic profiles at 14 time points across the C57/B6 mouse liver development, which include E11.5 (embryonic day 11.5), E12.5, E13.5, E14.5, E15.5, E16.5, E17.5, E18.5, Day0 (the day of birth), Day3, Day7, Day14, Day21, and normal adult liver. Orthologous genes were retrieved from both species, as previously described [19]. Clustering analysis based on mouse and human orthologous genes demonstrated that the gene expression profiles of differentiated HepaRG cells clustered with post-natal mouse samples, i.e., from Day0 to Day21 and adult liver samples (Figure 3A). Conversely, the gene expression profiles of HepaRG progenitor cells clustered with those of early embryonic livers, particularly between E11.5 and E14.5 (Figure 3A). Examples of the expression of developmentally regulated genes differentially expressed in differentiated and progenitor HepaRG cells (e.g., *PAH*, *HAO1*, *CXCR7*) are shown in Figure 3B.

### 3.4. HepaRG-Derived Signature Distinguishes Relevant Human HCC Subtypes

**Figure 4:**
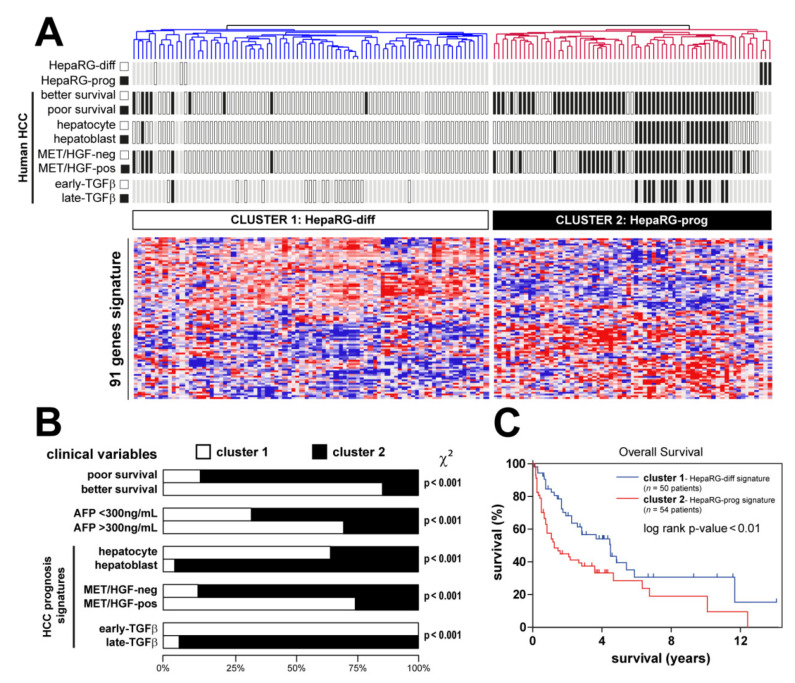
Clinical relevance of HepaRG signature in human HCC. (**A**) Dendrogram overview of in vitro experiments using differentiated and progenitor HepaRG cells (Figure 2) integrated with 139 cases of human HCC (NCI training set, Figure 1). Clustering analysis was based on the expression of genes differentially expressed between differentiated and progenitor HepaRG (Table S1). Two main clusters were identified: cluster 1 and cluster 2, associated with differentiated and progenitor HepaRG, respectively. Distribution of HCC samples between previously described subgroups with respect to survival (better vs. poor prognosis) [7], cell origin (hepatocyte vs. hepatoblast) [10], activation of MET/HGF (negative vs. positive) [29] and TGFβ signaling (early vs. late) [19] is indicated on the left side. (**B**) Statistical analysis of HCC distribution between clusters 1 and 2 based on previous gene expression signatures and clinical parameters. Cluster 2 (defined by a high expression of genes reflecting the HepaRG progenitor state) shows a significant enrichment in human HCC with the following features: poor survival, hepatoblast traits, activation of MET/HGF, late TGFβ pathways and higher serum AFP levels. (**C**) Kaplan–Meier plot and log-rank statistics analysis revealed a significant decrease in overall survival for patients included in cluster 2.

Based on the GSEA results demonstrating a significant enrichment of a signature featuring a well-differentiated human HCC subtype (Figure 2D), we hypothesized that the differentiation-associated signature established in HepaRG could be clinically relevant. To test this hypothesis, we integrated the HepaRG signature (Table S1) with the gene expression profiles of 139 cases of human HCC extensively characterized (NCI training dataset, Figure 1) [7,10,19,29]. Hierarchical clustering analysis of the integrated dataset (91 genes detected in both datasets) identified two major clusters (Figure 4A). Clusters 1 and 2 included HCC associated with differentiated and progenitor HepaRG, respectively. The biological and clinical parameters were not randomly distributed between the clusters (Figure 4A,B). Strikingly, cluster 1 included significantly more tumors, which were previously assigned to a better prognosis group, than cluster 2 (Figure 4A,B). Conversely, cluster 2 (progenitor HepaRG), was significantly enriched in HCC previously defined by poor prognosis signatures, including poor survival genes [7], hepatoblast-associated genes [10] and genes associated with the activation of a pro-oncogenic MET/HGF pathway [29] and a so-called late pro-metastatic TGFβ signature [19] (Figure 4A,B). The serum level of alpha-fetoprotein (AFP), a well-established HCC biomarker, was significantly higher in these aggressive HCC (Figure 4A,B). Accordingly, HCC linked to HepaRG progenitors exhibited a reduced OS (Figure 4C). Altogether, the data demonstrate that the progenitor HepaRG signature predicts a poor prognosis in human HCC.

### 3.5. A Minimal Subset of Seven Differentiation-Associated Genes Predicts a Poor Prognosis in Several Human Cancers

**Figure 5:**
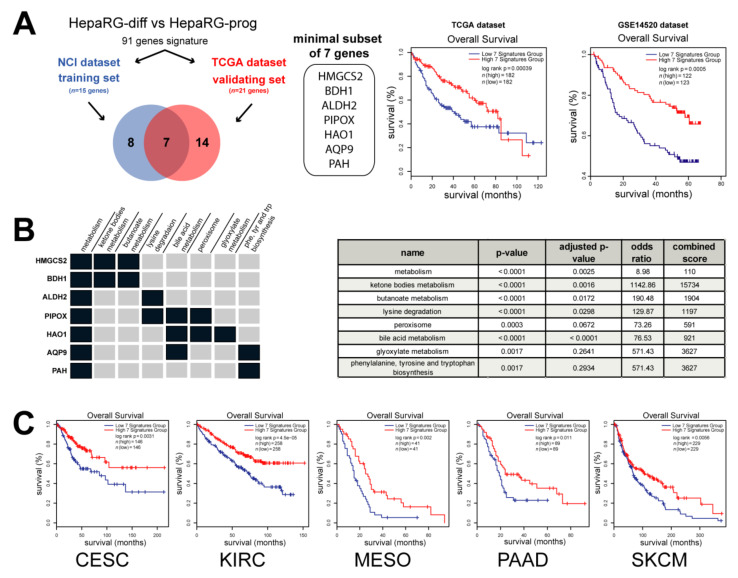
A minimal subset of 7 metabolic genes predicts a poor prognosis in cancer. (**A**) Analysis of genes from the HepaRG signature described in Figure 4 associated with the overall survival (OS) in two independent datasets: NCI (*n* = 15 genes) and TCGA (*n* = 21 genes). Survival analysis unraveled a minimal subset of 7 genes negatively correlated with patient outcome in two distinct datasets (i.e., TCGA, *n* = 364 HCC and GSE14520, *n* = 245 HCC). (**B**) Data mining analysis using Enrichr [30] demonstrated that the 7 genes were significantly associated with metabolism, such as ketone bodies, butanoate, bile acid and glyoxylate metabolisms, as well as lysine degradation, peroxisome and Phe, Tyr and Trp biosynthesis (*p* value < 0.01) (**C**) Analysis of overall survival by Kaplan–Meier plot and log-rank statistics in several cancers from TCGA, including cervical squamous cell carcinoma (CESC), kidney renal clear cell carcinoma (KIRC), mesothelioma (MESO), pancreatic adenocarcinoma (PAAD) and skin cutaneous melanoma (SKCM). Patients were divided into two groups based on the expression of the 7 genes taken as a signature (blue curve: low expression; red curve: high expression).

Next, from the clinically relevant differentiation-associated human gene expression signature, we selected a minimal subset of genes able to predict survival in independent cohorts of patients with HCC (Figure 1). From the NCI training dataset, 15 genes were associated with OS and 21 genes from the TCGA validating dataset, including seven common genes (Figure 5A). The expression of these seven genes (*HMGCS2*, *BDH1*, *ALDH2*, *PIPOX*, *HAO1*, *AQP9* and *PAH*) was negatively correlated with the survival of patients with HCC in two independent datasets (Figure 5A). In agreement with the selection process, data mining demonstrated that this minimal subset of genes was significantly enriched in metabolism (ketone bodies, butanoate, bile acid and glyoxylate metabolism, as well as lysine degradation, peroxisome and Phe, Tyr and Trp biosynthesis) (Figure 5B).

More interestingly, Kaplan–Meier plots and log-rank statistics demonstrated that a low expression of these seven metabolic genes (taken as a unique signature) predicted a decreased OS, not only in patients with HCC (Figure 5A), but also in patients with cervical squamous cell carcinoma, kidney renal clear cell carcinoma, mesothelioma, pancreatic adenocarcinoma and skin cutaneous melanoma (Figure 5C). Altogether, the data demonstrate that a minimal subset of seven metabolic genes associated with hepatocyte differentiation predicts a poor prognosis in several human cancers.

## 4. Discussion

In this study, by applying a functional and integrative transcriptomics approach based on the well-characterized HepaRG model of tumor hepatocyte differentiation, we identified a minimal subset of seven genes (*HMGCS2*, *BDH1*, *ALDH2*, *PIPOX*, *HAO1*, *AQP9* and *PAH*) predicting the survival of patients with cancer. These genes were mainly associated with metabolism, in agreement with metabolic reprogramming as a key hallmark of cancer cells [31]. All these genes were down-regulated in poor-prognosis HCC.

*HMGCS2* encodes the 3-hydroxy-3-methylglutaryl-CoA synthase 2, a mitochondrial enzyme that catalyzes the second and rate-limiting reaction of ketogenesis, a metabolic pathway that provides lipid-derived energy. In agreement with our findings, HMGCS2 was previously reported to be significantly down-regulated in HCC patients with high alpha-fetoprotein (AFP), tumor size >5 cm, multinodular, advanced tumor staging including BCLC, TNM and CLIP [32]. The Kaplan–Meier analysis also demonstrated that HMGCS2 down-regulation contributed to an unfavorable OS in liver [32] and prostate [33] cancer. HMGCS2 may function as a tumor suppressor. Indeed, its knockdown induces the proliferation and metastasis ability of HCC cells by enhancing c-Myc/cyclinD1 and epithelial-to-mesenchymal transition (EMT) signaling and alters lipid metabolism, resulting in increases in fatty acid, triglyceride, and cholesterol syntheses [34,35].

*BDH1* (3-hydroxybutyrate dehydrogenase 1) is also involved in the metabolism of ketones. *BDH1* was reported to be down-regulated in glioblastoma [36] and acute myeloid leukemia (AML) [37]. The down-regulation of *BDH1* in AML was notably associated with a worse prognosis [37]. In addition, *BDH1* knock-down promotes the viability and the proliferation of AML cells [37]. The down-regulation of both *HMGCS2* and *BDH1* suggests that the deregulation of ketone metabolism is a hallmark of poor prognosis tumors. Thus, impaired ketogenesis has been shown to contribute to abnormal glucose metabolism and to provoke steatohepatitis [38,39]. Accordingly, therapeutic ketogenic diets have the potential to lower glucose availability to cancer cells and have been associated with better outcomes [39]. It was also reported that ketone bodies inhibited the proliferation of pancreatic cancer cells and that a ketogenic diet reduced tumor growth in mice [40].

*ALDH2* (aldehyde dehydrogenase 2) is one of the major mitochondrial enzymes protecting cells from acetaldehyde toxicity. *ALDH2* down-regulation has been widely reported in HCC and associated with an unfavorable outcome [41,42]. The gain and loss of function experiments demonstrated that ALDH2 inhibited HCC cell migration and invasion both in vitro and in vivo by modulating the redox status of cells and by activating the AMP-activated protein kinase (AMPK) signaling pathway [41]. Recently, it was reported that ALDH2 deficiency promotes alcohol-associated liver cancer by activating oncogenic pathways via oxidized DNA-enriched extracellular vesicles [43].

*PIPOX* (pipecolic acid and sarcosine oxidase) encodes an enzyme participating in the pipecolate pathway (i.e., L-lysine degradation). PIPOX is a sarcosine-metabolizing enzyme that produces glycine from sarcosine, a potential oncometabolite. *PIPOX* has been poorly investigated in cancer, but its expression was particularly low in triple-negative breast tumors [44]. PIPOX was shown to reduce the oncogenic potential of prostate cells by metabolizing sarcosine and to be down-regulated in prostate cancer [45].

*HAO1* (hydroxy acid oxidase 1) is a liver-specific peroxisomal enzyme that oxidizes glycolate to glyoxylate. *HAO1* has been linked to primary hyperoxaluria type 1. Supporting our gene signature, a recent study demonstrated that *HAO1* is down-regulated in HCC and that a low expression of *HAO1* predicts a reduced OS [46].

*AQP9* (aquaporin 9) has been extensively reported in cancer but with contrasting observations. Thus, a high expression of *AQP9* was significantly correlated with a worse prognosis in breast, colon and lung cancers, while predicted a better prognosis in gastric cancer [47]. In agreement with our results, *AQP9* was reported to be down-regulated in HCC tissues and human hepatoma cell lines in several studies and associated with a worse prognosis [48,49]. *AQP9* over-expression was shown to suppress the invasion of hepatoma cell by inhibiting EMT [48] and to inhibit the growth of subcutaneously xenografted liver tumors in nude mice [50].

*PAH* (phenylalanine hydroxylase, an enzyme which catalyzes the conversion of phenylalanine to tyrosine) has been associated with phenylketonuria, an autosomal recessive metabolic disorder. In HCC, a low expression of *PAH* has been reported as a prognostic marker for a poor prognosis [51].

Altogether, the data from the literature reporting the function of our minimal subset of seven genes predicting the survival of patients with HCC fully support a down-regulation in poor prognosis tumors. However, it has to be noted that the study exhibits some limitations. Notably, it is based on the gene expression profiling of a single HCC cell line. Due to the complexity of the liver structure, simplified in vitro models do not adequately reflect in vivo situations, especially the spatial heterogeneity and the metabolic zonation. Therefore, one can expect that further investigations using in vivo models would also be relevant to identify biomarkers related to hepatocyte differentiation. In addition, most of the current biomarkers are detected at the protein level. Thus, the detection of seven proteins at the same time by immunohistochemistry could be challenging. However, the detection of the gene signature at the protein level is not mandatory since alternative approaches already exist to assess complex gene expression signatures at the mRNA level in tissue biopsies. For instance, a six-gene signature has been recently established to accurately predict microvascular invasion in human HCC. These genes were identified by screening for the expression of 200 genes using a NanoString system in more than 300 frozen and/or FFPE samples [52]. The authors concluded that the approach could be applied in clinical practice with a routine tumor biopsy and integrated into therapeutic strategies [52].

## 5. Conclusions

In conclusion, our study identified a subset of seven genes reflecting the differentiation status of tumor hepatocytes. This minimal signature is clinically relevant for predicting the prognosis of HCC patients and can be easily implemented. Although these genes do not encode secreted proteins, it would be interesting to determine whether the products of the corresponding impaired metabolisms could be detected in the serum of the patients using poorly invasive methods and predictive of the clinical outcome.

## Data Availability

The data presented in this study are openly available from the gene expression omnibus database (https://www.ncbi.nlm.nih.gov/geo, accessed on July 2021).

## Supplementary Materials

The following are available online at https://www.mdpi.com/article/10.3390/cancers13225624/s1, Table S1: List of genes differentially expressed between differentiated and progenitor HepaRG cells.
