# Supplementary material for: A Minimal Subset of Seven Genes Associated with Tumor Hepatocyte Differentiation Predicts a Poor Prognosis in Human Hepatocellular Carcinoma
Source: Cancers (Basel). 2021 Nov 10;13(22):5624. doi: 10.3390/cancers13225624 (PMC8616205; doi:10.3390/cancers13225624)
Supplement: Supplementary file 1 [file cancers-13-05624-s001.zip › cancers-1425904-supplementary.pdf]

Supplementary Table S1. List of genes differentially expressed between differentiated and progenitor HepaRG cells

A) Genes up-regulated in differentiated HepaRG cells

Probe ID	Gene symbol	Expression value in differentiated HepaRG	Expression value in progenitor HepaRG	Fold-change FC differentiated/ progenitor	Parametric p-value	Log2 (FC)	Log10 (p-value)
A_23_P116898	A2M	1.34	0.58	2.32	0.0008	1.21	3.12
A_33_P3361112	ABCG8	8.87	0.32	27.35	0.0008	4.77	3.08
A_23_P376727	ACMSD	2.1	0.34	6.14	0.0002	2.62	3.70
A_24_P291658	ADH1A	6.84	0.45	15.06	0.0002	3.91	3.60
A_23_P149926	AKR1C4	2.1	0.3	6.96	0.0005	2.80	3.34
A_23_P36753	ALDH2	1.3	0.64	2.04	0.0006	1.03	3.25
A_24_P353619	ALPL	1.18	0.37	3.15	0.0002	1.66	3.61
A_33_P3295358	ANGPTL4	1.79	0.072	24.97	0.0009	4.64	3.04
A_23_P60009	ANXA13	6.45	0.42	15.27	0.0008	3.93	3.12
A_23_P417974	AQP11	2.28	0.72	3.16	0.0001	1.66	3.87
A_23_P106362	AQP9	1.28	0.27	4.78	0.0002	2.26	3.66
A_33_P3252359	BDH1	8.82	0.21	41.44	0.0001	5.37	4.23
A_33_P3396886	C11orf52	2	0.9	2.23	0.0002	1.16	3.62
A_32_P162187	C2	1.12	0.39	2.85	0.0005	1.51	3.34
A_23_P42282	C4B	1.12	0.55	2.05	0.0002	1.04	3.69
A_33_P3284508	CD14	1.09	0.4	2.7	0.0002	1.43	3.70
A_23_P206359	CDH1	2.91	0.46	6.33	0.0008	2.66	3.11
A_33_P3268507	CEACAM1	1.23	0.38	3.23	0.0007	1.69	3.18
A_23_P156687	CFB	1.01	0.31	3.23	0.0000	1.69	5.89
A_23_P114740	CFH	1.27	0.45	2.82	0.0001	1.50	3.90
A_24_P92472	CFI	1.38	0.65	2.11	0.0000	1.08	4.34
A_23_P69293	CHDH	1.75	0.78	2.26	0.0002	1.18	3.60
A_23_P141035	CHST4	2.08	0.67	3.12	0.0002	1.64	3.76
A_33_P3249046	CLDN2	3.14	0.4	7.92	0.0000	2.99	5.14
A_33_P3242798	CPS1	2.18	0.52	4.17	0.0003	2.06	3.57
A_33_P3281985	CR2	3.65	0.17	21.49	0.0005	4.43	3.34
A_33_P3317670	CRP	1.42	0.058	24.38	0.0009	4.61	3.04
A_23_P163402	CYP1A1	9.85	0.79	12.46	0.0000	3.64	4.99
A_24_P208704	CYP8B1	3.86	0.11	34.11	0.0000	5.09	4.38
A_24_P10233	DAPK2	1.96	0.55	3.57	0.0004	1.84	3.44

A_33_P3240163	DEFB109P1B	1.58	0.3	5.29	0.0002	2.40	3.74
A_32_P175301	DENND3	1.82	0.9	2.03	0.0001	1.02	4.05
A_23_P15202	DHODH	1.27	0.55	2.31	0.0002	1.21	3.80
A_23_P139704	DUSP6	2.68	0.55	4.88	0.0005	2.29	3.34
A_33_P3365750	EML5	2.45	0.9	2.71	0.0009	1.44	3.04
A_33_P3420224	ENTPD8	2.79	0.41	6.77	0.0002	2.76	3.72
A_23_P375372	FGA	1.16	0.23	4.95	0.0002	2.31	3.80
A_23_P44274	FGA	1.14	0.26	4.32	0.0002	2.11	3.78
A_23_P500501	FGFR3	1.79	0.64	2.81	0.0003	1.49	3.58
A_23_P148088	FGG	1.08	0.21	5.21	0.0001	2.38	4.11
A_23_P128246	FICD	1.41	0.54	2.61	0.0007	1.38	3.15
A_23_P8497	GHRHR	1.62	0.52	3.12	0.0009	1.64	3.03
A_24_P404840	GJB1	3.39	0.22	15.55	0.0002	3.96	3.72
A_23_P75749	GLYAT	5.98	0.81	7.35	0.0001	2.88	3.93
A_23_P370666	GLYATL1	5.34	0.32	16.6	0.0002	4.05	3.61
A_33_P3369371	GPX3	1.4	0.55	2.55	0.0008	1.35	3.08
A_23_P133474	GPX3	1.24	0.51	2.42	0.0001	1.28	4.26
A_23_P29257	H1F0	1.18	0.54	2.19	0.0001	1.13	3.97
A_23_P154708	HAO1	2.86	0.29	9.84	0.0003	3.30	3.50
A_23_P103588	HMGCS2	3.37	0.94	3.59	0.0003	1.84	3.48
A_23_P206760	HP	1.14	0.11	10.16	0.0000	3.34	6.52
A_33_P3289236	HPR	1.48	0.06	24.89	0.0003	4.64	3.57
A_23_P252306	ID1	1.37	0.16	8.58	0.0002	3.10	3.79
A_23_P62607	IL22RA1	1.23	0.55	2.23	0.0006	1.16	3.24
A_24_P141707	INHBE	2.88	0.43	6.71	0.0000	2.75	4.87
A_33_P3260373	ISY1-RAB43	1.17	0.54	2.17	0.0008	1.12	3.11
A_23_P128084	ITGA7	1.96	0.43	4.58	0.0010	2.20	3.01
A_23_P319423	KCNK5	2.12	0.53	3.97	0.0008	1.99	3.10
A_32_P46594	LOC145837	3.03	0.44	6.9	0.0002	2.79	3.64
A_23_P50638	LRG1	1.06	0.21	5.19	0.0000	2.38	4.63
A_23_P103110	MAFF	1.17	0.27	4.34	0.0002	2.12	3.74
A_23_P23996	MAT1A	2.33	0.22	10.5	0.0005	3.39	3.34
A_33_P3215457	MTTP	2.33	0.33	7.04	0.0008	2.82	3.10
A_23_P328740	NEURL3	2.18	0.45	4.85	0.0005	2.28	3.30
A_33_P3406866	NFASC	1.91	0.75	2.56	0.0008	1.36	3.11
A_23_P41804	NKD2	2.17	0.58	3.75	0.0010	1.91	3.02
A_23_P160800	NR0B2	2.06	0.56	3.67	0.0003	1.88	3.47
A_23_P319572	NR1I3	1.76	0.5	3.51	0.0007	1.81	3.16
A_33_P3335057	OR8U8	2.83	0.75	3.75	0.0010	1.91	3.01

A_23_P169494	ORM1	1.3	0.34	3.88	0.0009	1.96	3.04
A_23_P2501	PAH	9.58	0.24	40.74	0.0007	5.35	3.14
A_23_P35977	PDZD3	4.46	0.87	5.14	0.0001	2.36	4.27
A_33_P3259917	PGLYRP2	1.48	0.54	2.74	0.0006	1.45	3.20
A_23_P210465	PI3	1.13	0.51	2.22	0.0001	1.15	4.02
A_23_P164258	PIPOX	2.13	0.28	7.67	0.0001	2.94	4.01
A_23_P388150	PLA2G12B	7.97	0.045	178.86	0.0002	7.48	3.81
A_23_P50508	PLA2G4C	1.06	0.45	2.32	0.0000	1.21	4.46
A_32_P66364	PPP1R1C	1.76	0.84	2.09	0.0006	1.06	3.23
A_33_P3379922	PROC	1.89	0.76	2.48	0.0000	1.31	4.95
A_23_P165201	PRODH2	2.06	0.28	7.31	0.0002	2.87	3.68
A_23_P118392	RASD1	1.88	0.12	15.53	0.0002	3.96	3.74
A_24_P218814	RDH5	2.27	0.93	2.43	0.0007	1.28	3.15
A_23_P320578	RGS16	2.02	0.79	2.54	0.0001	1.34	4.28
A_33_P3408918	SAA2	1.03	0.29	3.53	0.0001	1.82	4.06
A_23_P87238	SAA4	1.53	0.039	38.92	0.0007	5.28	3.14
A_33_P3289659	SERPINA1	1.24	0.086	14.31	0.0006	3.84	3.19
A_23_P2920	SERPINA3	1.33	0.13	9.96	0.0006	3.32	3.21
A_33_P3399788	SERPINA3	1.32	0.14	9.31	0.0010	3.22	3.01
A_33_P3413989	SERPING1	1.35	0.21	6.58	0.0008	2.72	3.09
A_33_P3413987	SERPING1	1.28	0.22	5.83	0.0008	2.54	3.10
A_32_P205624	SHC2	1.28	0.57	2.24	0.0010	1.16	3.02
A_23_P42189	SLC17A1	7.13	0.096	74.39	0.0010	6.22	3.01
A_33_P3379926	SLC17A4	3.98	0.2	19.8	0.0000	4.31	4.62
A_33_P3409154	SLC22A23	1.09	0.42	2.57	0.0003	1.36	3.50
A_23_P422511	SLC25A45	1.62	0.57	2.84	0.0008	1.51	3.11
A_23_P64070	SLC25A45	1.68	0.6	2.8	0.0002	1.49	3.64
A_24_P179816	SLC27A3	2.48	0.95	2.62	0.0002	1.39	3.72
A_24_P268685	SLC6A1	9.56	0.2	46.66	0.0002	5.54	3.61
A_23_P131846	SNAI1	1.16	0.24	4.86	0.0004	2.28	3.42
A_23_P28186	SRD5A2	5.08	0.14	37.12	0.0009	5.21	3.02
A_23_P382188	STAP2	1.17	0.4	2.95	0.0001	1.56	3.90
A_23_P80974	TDO2	3.62	0.22	16.76	0.0003	4.07	3.50
A_23_P108157	TJP3	2.43	0.73	3.33	0.0001	1.74	4.09
A_33_P3230219	TMEM54	2.01	0.83	2.42	0.0003	1.28	3.48
A_23_P29067	TMPRSS2	1.52	0.35	4.39	0.0004	2.13	3.38
A_33_P3226810	TNFSF10	0.96	0.24	4.02	0.0001	2.01	4.29
A_23_P121253	TNFSF10	0.92	0.24	3.82	0.0005	1.93	3.35
A_24_P237036	TNFSF14	1.2	0.5	2.38	0.0009	1.25	3.03

A_23_P386912	UGT2B4	4.04	0.16	25.87	0.0002	4.69	3.70
A_19_P00809915	XLOC_006829	2.68	0.92	2.92	0.0001	1.55	3.91
A_23_P250619	ZDHHC14	1.43	0.56	2.56	0.0000	1.36	5.46
A_24_P269129	ZDHHC14	1.27	0.63	2.01	0.0004	1.01	3.45
A_33_P3372413	nd	2.39	0.17	14.18	0.0003	3.83	3.59
A_33_P3300495	nd	2.86	0.54	5.27	0.0008	2.40	3.08
A_32_P153979	nd	1.11	0.33	3.33	0.0009	1.74	3.05
A_33_P3240295	nd	2.56	0.92	2.8	0.0002	1.49	3.62
A_19_P00326161	nd	1.03	0.41	2.51	0.0009	1.33	3.06
A_33_P3227556	nd	1.4	0.57	2.45	0.0007	1.29	3.13

B) Genes down-regulated in differentiated HepaRG cells

Probe ID	Gene symbol	Expression value in differentiated HepaRG	Expression value in progenitor HepaRG	Fold-change FC differentiated/ progenitor	Parametric p-value	Log2 (FC)	Log10 (p-value)
A_23_P127948	ADM	0.87	2.16	0.4	0.0009	-1.32	3.05
A_23_P379649	BMF	0.27	3.08	0.089	0.0002	-3.49	3.62
A_33_P3284813	C10orf113	0.6	3.02	0.2	0.0007	-2.32	3.18
A_33_P3312119	C6orf99	0.18	5.6	0.032	0.0003	-4.97	3.52
A_33_P3218960	CACNA1H	0.8	1.66	0.49	0.0003	-1.03	3.59
A_23_P21092	CALB2	0.65	2.06	0.32	0.0007	-1.64	3.14
A_33_P3367341	CASP7	0.26	1.03	0.25	0.0005	-2.00	3.27
A_33_P3287825	CCDC136	0.88	1.83	0.48	0.0000	-1.06	4.88
A_23_P62764	CCDC28B	0.68	1.52	0.45	0.0001	-1.15	4.11
A_23_P20752	CDK20	0.99	2.23	0.44	0.0005	-1.18	3.27
A_33_P3213374	CITED2	0.93	2.28	0.41	0.0004	-1.29	3.45
A_23_P205031	COL4A2	0.56	1.22	0.45	0.0001	-1.15	4.30
A_33_P3227400	COL4A4	0.41	2.06	0.2	0.0002	-2.32	3.70
A_23_P83818	COL5A1	0.34	1.48	0.23	0.0002	-2.12	3.65
A_23_P7144	CXCL1	0.42	1.24	0.34	0.0007	-1.56	3.14
A_23_P131676	CXCR7	0.53	2.35	0.22	0.0002	-2.18	3.68
A_23_P152262	DPEP1	0.24	2.72	0.089	0.0008	-3.49	3.12
A_33_P3251841	DSEL	0.91	3.04	0.3	0.0005	-1.74	3.27

A_23_P401606	EDIL3	0.66	2.36	0.28	0.0001	-1.84	4.30
A_33_P3404749	FMN1	0.71	2.26	0.31	0.0000	-1.69	4.47
A_23_P26037	FRMD5	0.88	2.06	0.43	0.0008	-1.22	3.12
A_32_P74579	GAS2L2	0.51	4.06	0.13	0.0009	-2.94	3.07
A_24_P227069	GPAM	0.33	1.53	0.21	0.0001	-2.25	3.94
A_33_P3340639	GPAM	0.25	1.91	0.13	0.0000	-2.94	4.61
A_19_P00330814	HOTAIR	0.12	1.96	0.06	0.0000	-4.06	5.80
A_33_P3338698	IHH	0.44	1.46	0.3	0.0003	-1.74	3.48
A_23_P15786	KRT25	0.22	1.45	0.15	0.0002	-2.74	3.81
A_33_P3292886	KRT6A	0.56	1.62	0.35	0.0005	-1.51	3.34
A_33_P3287800	LBX2	0.45	1.35	0.33	0.0005	-1.60	3.34
A_23_P6771	LMCD1	0.48	1.26	0.38	0.0003	-1.40	3.49
A_33_P3369436	LOC100130111	0.8	1.94	0.41	0.0004	-1.29	3.37
A_33_P3285145	LOC100131826	0.58	1.97	0.29	0.0010	-1.79	3.01
A_24_P306594	LOC100506310	0.52	1.66	0.32	0.0001	-1.64	3.96
A_33_P3474859	LOC203274	0.82	1.71	0.48	0.0005	-1.06	3.32
A_32_P63013	LOC283174	0.14	4.24	0.033	0.0002	-4.92	3.62
A_19_P00318599	LOC648987	0.63	1.34	0.47	0.0007	-1.09	3.17
A_33_P3364741	MRC2	0.14	1.38	0.1	0.0002	-3.32	3.75
A_23_P148737	MYBPH	0.83	4.18	0.2	0.0003	-2.32	3.47
A_23_P65278	NBEA	0.86	1.96	0.44	0.0004	-1.18	3.46
A_24_P135322	NRP1	0.81	1.85	0.44	0.0009	-1.18	3.03
A_23_P348257	NUAK1	0.39	1.48	0.26	0.0001	-1.94	3.88
A_24_P229531	OBFC2A	0.67	1.55	0.43	0.0004	-1.22	3.39
A_24_P299474	ODZ2	0.74	2.86	0.26	0.0006	-1.94	3.26
A_33_P3599591	PAPPA	0.64	1.68	0.38	0.0003	-1.40	3.49
A_23_P420236	PCDH9	0.82	3.35	0.24	0.0001	-2.06	3.92
A_33_P3378514	PDE5A	0.87	6.08	0.14	0.0001	-2.84	4.03
A_33_P3282162	PLAC4	0.67	1.68	0.4	0.0010	-1.32	3.02
A_23_P17914	PNPLA3	0.7	1.59	0.44	0.0007	-1.18	3.17
A_33_P3511265	POSTN	0.64	1.98	0.33	0.0002	-1.60	3.76
A_24_P916496	PRKCA	0.86	2.24	0.38	0.0006	-1.40	3.21
A_33_P3365087	PRR20B	0.4	3.34	0.12	0.0004	-3.06	3.35

A_33_P3294917	PRUNE2	0.59	1.92	0.31	0.0002	-1.69	3.75
A_33_P3218832	RIMS1	0.81	2.24	0.36	0.0007	-1.47	3.14
A_23_P216307	RUNX1T1	0.51	4.68	0.11	0.0001	-3.18	3.94
A_33_P3269636	SBSN	0.46	2.86	0.16	0.0007	-2.64	3.14
A_24_P760960	SKINTL	0.14	1.81	0.075	0.0007	-3.74	3.13
A_33_P3277447	SLC26A2	0.76	2.1	0.36	0.0005	-1.47	3.31
A_33_P3331906	SNTB2	0.82	1.84	0.45	0.0000	-1.15	4.45
A_33_P3281333	SNTB2	0.78	1.95	0.4	0.0002	-1.32	3.65
A_33_P3382924	SPARC	0.43	1.4	0.31	0.0006	-1.69	3.24
A_23_P431388	SPOCD1	0.41	2.25	0.18	0.0010	-2.47	3.00
A_32_P134968	SPTB	0.89	2.41	0.37	0.0008	-1.43	3.08
A_32_P98072	TCHH	0.54	2.51	0.22	0.0001	-2.18	4.03
A_33_P3345210	TLCD2	0.68	1.38	0.49	0.0005	-1.03	3.28
A_23_P101392	TMEM38A	1.01	2.13	0.47	0.0000	-1.09	4.33
A_32_P2452	TMTC1	0.6	1.38	0.43	0.0008	-1.22	3.10
A_23_P94403	TYRP1	0.79	1.97	0.4	0.0007	-1.32	3.15
A_33_P3279629	UCN2	0.44	1.72	0.26	0.0002	-1.94	3.66
A_23_P102611	WISP2	0.45	1.58	0.28	0.0009	-1.84	3.06
A_19_P00318909	XLOC_l2_010751	0.85	1.97	0.43	0.0000	-1.22	4.55
A_19_P00321221	XLOC_l2_011969	0.99	2.52	0.39	0.0007	-1.36	3.15
A_19_P00813148	nd	0.95	2.02	0.47	0.0005	-1.09	3.28
A_33_P3415199	nd	0.96	2.3	0.42	0.0010	-1.25	3.02
A_33_P3271341	nd	0.65	1.67	0.39	0.0000	-1.36	4.65
A_19_P00321544	nd	0.9	2.43	0.37	0.0002	-1.43	3.61
A_24_P691826	nd	0.66	1.91	0.35	0.0006	-1.51	3.21
A_19_P00324423	nd	0.87	2.59	0.34	0.0002	-1.56	3.77
A_33_P3401482	nd	0.28	1.21	0.23	0.0003	-2.12	3.58
A_33_P3279193	nd	0.25	5	0.051	0.0003	-4.29	3.50
